# The Experimental TASK-1 Potassium Channel Inhibitor A293 Can Be Employed for Rhythm Control of Persistent Atrial Fibrillation in a Translational Large Animal Model

**DOI:** 10.3389/fphys.2021.668267

**Published:** 2021-04-12

**Authors:** Hélène Le Ribeuz, David Montani, Fabrice Antigny

**Affiliations:** ^1^Université Paris-Saclay, Faculté de Médecine, Le Kremlin-Bicêtre, France; ^2^INSERM UMR_S 999 ≪ Hypertension Pulmonaire: Physiopathologie et Innovation Thérapeutique ≫, Hôpital Marie Lannelongue, Le Plessis-Robinson, France; ^3^Assistance Publique - Hôpitaux de Paris (AP-HP), Service de Pneumologie et Soins Intensifs Respiratoires, Centre de Référence de l'Hypertension Pulmonaire, Hôpital Bicêtre, Le Kremlin-Bicêtre, France

**Keywords:** TASK-1, KCNK3, pulmonary hypertension, atrial fibrillation, A293

## Introduction

The authors report in a porcine model of persistent atrial fibrillation (AF) that long term *in vivo* pharmacological inhibition of TASK-1 potassium channels with A293 compound (1 mg/kg/day) has antiarrhythmic effects, suggesting that TASK-1 inhibition can be used to counteract rhythm abnormalities in AF patients (Wiedmann et al., 2020b). Previously, the same group, using *in vitro* experiments described an upregulation of TASK-1 in patients with AF, which was associated with a shortening of the human atrial action potential (Schmidt et al., 2015). Moreover, using isolated human and porcine atrial cardiomyocytes, they also demonstrated that pharmacological inhibition of atrial TASK-1 induces antiarrhythmic effects *in vitro* as well as *in silico*. They found that short-term *in vivo* pharmacological inhibition of TASK-1 by A293 (1 mg/Kg/day) does not alter the surface ECG parameters of healthy control pigs (Wiedmann et al., 2020a). Using A293, they also showed that pharmacological inhibition of TASK-1 prolongs action potential duration in atrial myocytes obtained from patients with chronic atrial fibrillation *in vitro* (Schmidt et al., 2015). Undoubtedly, all these results and the fact that mutations were found in the *KCNK3* gene (coding TASK-1 channel) from some patients with AF (Liang et al., 2014) clearly demonstrated that TASK-1 is crucially involved in AF, and clearly indicate that this potassium channel should be an interesting therapeutic target in AF.

## Subsections Relevant for the Subject

However, TASK-1 is expressed in several cell types, including pulmonary arterial smooth muscle cells (PASMCs) and pulmonary arterial endothelial cells (PAECs), in human, pigs and rats. Moreover, since 2013, 12 different *KCNK3* mutations have been identified in at least 19 patients from different cohorts of patients with pulmonary arterial hypertension (PAH) (Le Ribeuz et al., 2020a). Heritable PAH due to *KCNK3* mutations is characterized by autosomal dominant inheritance with incomplete penetrance (Morrell et al., 2019). To date, all *KCNK3* mutations analyzed by whole-cell patch-clamp analysis have led to loss-of-function (LOF) of the T*ASK-1* channel (Le Ribeuz et al., 2020a), indicating that TASK-1 LOF at least predisposes to the development of PAH. Indeed, previous studies showed that TASK-1 inactivation by siRNA or pharmacological inhibition (A293 compound) in human PASMCs (hPASMCs) and rat PASMCs leads to the depolarization of resting membrane potential (Olschewski et al., 2006; Antigny et al., 2016). We also found *in vitro* in hPASMCs that TASK-1 LOF (siRNA or inhibition with A293) enhances the proliferation of hPASMCs (Lambert et al., 2019). In control hPAECs and hPASMCs we recently found that the loss of TASK-1 expression by a specific siRNA induces deregulation of several signaling pathways involved in the control of cell proliferation, cell migration, cell apoptosis, and cell metabolism (Le Ribeuz et al., 2020b). In association with PASMC depolarization, we found that TASK-1 inhibition with A293 or *Task-1*-LOF-mutation causes pulmonary artery vasoconstriction in rats (Antigny et al., 2016; Lambert et al., 2019).

Additionally, we found that *in vivo* inhibition of TASK-1 in rats with A293 at 10 mg/kg/day induced the development of significant early signs of PAH, with abnormal elevation of right ventricular systolic pressure (RVSP), abnormal pulmonary vascular cell proliferation, pulmonary vessel remodeling, and lung inflammation (Antigny et al., 2016) as well as RV hypertrophy, RV fibrosis, RV inflammation, and a subsequent decrease in RV performance (Lambert et al., 2018). Using *Kcnk3*-LOF mutated rats, we recently confirmed that TASK-1 LOF is a key event in PAH pathogenesis, promoting the elevation of RVSP, distal lung neomuscularization, perivascular extracellular matrix activation, overactivation of proliferative and survival signaling pathways and alteration of RV cardiomyocyte excitability (Lambert et al., 2019). Importantly, in contrast to other cell types, TASK-1 was the unique TASK channel expressed in the pulmonary vasculature (Olschewski et al., 2017) making pulmonary vascular cells more susceptible to TASK-1 channel inhibition than other tissues.

## Discussion

In line with these results, Wiedmann et al., found that chronic *in vivo* inhibition of TASK-1 (for 14 days) in a porcine model of persistent AF was associated with an increase in pulmonary arterial pressure (Wiedmann et al., 2020b), confirming that TASK-1 is a crucial channel for the maintenance of pulmonary vasculature homeostasis. Indeed, the TASK-1 channel contributes to the resting membrane potential of several additional cells, including neurons, carotid bodies, and in adrenal glands. Regarding the role of TASK-1 in aldosterone production in mice (Heitzmann et al., 2008; Chen et al., 2015), the measurement of aldosterone level in “AF-like pigs” treated with A293 may be informative. In pancreatic α-cell, TASK-1 inhibition modulates glucose-dependent inhibition of glucagon secretion by regulating the α-cell excitability (Dadi et al., 2015), in patients potentially treated with TASK-1 inhibitors glycaemia should be regularly assessed. Indeed, as TASK-1 channel plays a role in the chemosensory control of breathing in mice (Bayliss et al., 2015; Buehler et al., 2017), it would be of interest to report if the authors have noticed any effects on animal respiration or gas exchanges. Additionally, TASK-1 is functionally expressed in T lymphocytes, contributing to outward-K^+^ currents, and the *in vitro* and in vivo selective TASK1 inhibition reduces the T cell proliferation and cytokine production (Meuth et al., 2008; Bittner et al., 2012; Bittner and Meuth, 2013), which could have profound consequence for autoimmune response in patients treated with TASK-1 channels blockers. Finally, in mice the knockdown of TASK-1 significantly decreased the formation of blastocyst by 38% suggesting that TASK-1 is required for mouse embryonic development (Hur et al., 2012). To avoid any risk during embryonic development, TASK-1 blockers administration should not be administrated to women who are or wish to be pregnant.

For all these physiological role played by TASK-1 in several tissues, the *in vivo* pharmacological inhibition of TASK-1 should be extremely managed in treated-patients with AF.

Based on these results, one can hypothesize that A293 may induce PAH and right ventricular dysfunction in humans. Given the long history of drug-induced PAH [anorexigens (Montani et al., 2013), tyrosine kinase inhibitors (Weatherald et al., 2017), and chemotherapy (Certain et al., 2020)], we wish to convey the need for the close monitoring and screening echocardiography for PAH in patients chronically treated with TASK-1 inhibitors.

## Author Contributions

HL, DM, and FA drafted the manuscript. All authors reviewed and revised the final version and approved manuscript submission.

## Conflict of Interest

The authors declare that the research was conducted in the absence of any commercial or financial relationships that could be construed as a potential conflict of interest.
